# Long-term lithium treatment in bipolar disorder: effects on glomerular filtration rate and other metabolic parameters

**DOI:** 10.1186/s40345-017-0096-2

**Published:** 2017-08-01

**Authors:** Leonardo Tondo, Maria Abramowicz, Martin Alda, Michael Bauer, Alberto Bocchetta, Lorenza Bolzani, Cynthia V. Calkin, Caterina Chillotti, Diego Hidalgo-Mazzei, Mirko Manchia, Bruno Müller-Oerlinghausen, Andrea Murru, Giulio Perugi, Marco Pinna, Giuseppe Quaranta, Daniela Reginaldi, Andreas Reif, Philipp Ritter, Janusz K. Rybakowski, David Saiger, Gabriele Sani, Valerio Selle, Thomas Stamm, Gustavo H. Vázquez, Julia Veeh, Eduard Vieta, Ross J. Baldessarini

**Affiliations:** 1 Department of Psychiatry, Harvard Medical School, Boston, MA USA; 2 The International Consortium for Mood & Psychotic Disorders Research, MRC 306, McLean Hospital, 115 Mill Street, Belmont, MA 02478-9106 USA; 3 Lucio Bini Mood Disorders Centers, Cagliari and Rome, Italy; 4 Department of Adult Psychiatry, Poznan University of Medical Sciences, Poznan, Poland; 5 Department of Psychiatry, Dalhousie University, Halifax, NS Canada; 6 Department of Psychiatry and Psychotherapy, University Hospital Carl Gustav Carus, Technische Universität Dresden, Dresden, Germany; 7 Department of Biomedical Sciences, University of Cagliari, Cagliari, Italy; 8 Unit of Clinical Pharmacology, Azienda Ospedaliero-Universitaria, Cagliari, Italy; 9 Viarnetto Psychiatric Clinic, Lugano, Switzerland; 10 Hospital Clinic, University of Barcelona, IDIBAPS, CIBERSAM, Barcelona, Spain; 11 Section of Psychiatry, Department of Medical Science and Public Health, University of Cagliari, Cagliari, Italy; 12 Department of Pharmacology, Dalhousie University, Halifax, NS Canada; 13 Freie Universität Berlin, Charité Universitäts-Medizin, Berlin, Germany; 14 Department of Experimental and Clinic Medicine, Section of Psychiatry, University of Pisa & Institute of Behavioral Science, Pisa, Italy; 15 Department of General Psychology, Clinical Psychology, University of Padua, Padua, Italy; 16 Department of Psychiatry, Psychosomatic Medicine and Psychotherapy, University Hospital Frankfurt, Frankfurt-am-Main, Germany; 17 Department of Psychiatry, Charité Universitäs-Medizin, Berlin, Germany; 18 Brandenburg Medical School, Neureppin, Germany; 19 NeSMOS Department, ‘Sapienza’ University and Lucio Bini Mood Disorders Center, Rome, Italy; 20 Department of Psychiatry, Queens University, Kingston, Ontario Canada; 21 Department of Neuroscience, Palermo University, Buenos Aires, Argentina

**Keywords:** Blood urea nitrogen, Body-mass index, Creatinine, eGFR, Glomerular filtration rate, Glucose, Lithium, Staging of renal function, White blood cell count

## Abstract

**Background:**

Concerns about potential adverse effects of long-term exposure to lithium as a mood-stabilizing treatment notably include altered renal function. However, the incidence of severe renal dysfunction; rate of decline over time; effects of lithium dose, serum concentration, and duration of treatment; relative effects of lithium exposure vs. aging; and contributions of sex and other factors all remain unclear.

**Methods:**

Accordingly, we acquired data from 12 collaborating international sites and 312 bipolar disorder patients (6142 person-years, 2669 assays) treated with lithium carbonate for 8–48 (mean 18) years and aged 20–89 (mean 56) years. We evaluated changes of estimated glomerular filtration rate (eGFR) as well as serum creatinine, urea–nitrogen, and glucose concentrations, white blood cell count, and body-mass index, and tested associations of eGFR with selected factors, using standard bivariate contrasts and regression modeling.

**Results:**

Overall, 29.5% of subjects experienced at least one low value of eGFR (<60 mL/min/1.73 m^2^), most after ≥15 years of treatment and age > 55; risk of ≥2 low values was 18.1%; none experienced end-stage renal failure. eGFR declined by 0.71%/year of age and 0.92%/year of treatment, both by 19% more among women than men. Mean serum creatinine increased from 0.87 to 1.17 mg/dL, BUN from 23.7 to 33.1 mg/dL, glucose from 88 to 122 mg/dL, and BMI from 25.9 to 26.6 kg/m^2^. By multivariate regression, risk factors for declining eGFR ranked: longer lithium treatment, *lower* lithium dose, higher serum lithium concentration, older age, and medical comorbidity. Later low eGFR was also predicted by lower initial eGFR, and starting lithium at age ≥ 40 years.

**Limitations:**

Control data for age-matched subjects not exposed to lithium were lacking.

**Conclusions:**

Long-term lithium treatment was associated with gradual decline of renal functioning (eGFR) by about 30% more than that was associated with aging alone. Risk of subnormal eGFR was from 18.1% (≥2 low values) to 29.5% (≥1 low value), requiring about 30 years of exposure. Additional risk factors for low eGFR were higher serum lithium level, longer lithium treatment, lower initial eGFR, and medical comorbidity, as well as older age.

## Background

Decades of widespread international clinical use of lithium salts with controlled dosing, as well as extensive therapeutic research, support the value of lithium as a cornerstone of long-term, prophylactic treatment of patients diagnosed with bipolar disorder (Bauer et al. 2006; Baldessarini 2013; Severus et al. 2014; Bauer and Gitlin 2016). Nevertheless, an adverse effect of major concern associated with long-term lithium treatment is the risk of developing chronic kidney disease (CKD). This outcome usually is defined as a decrease of glomerular filtration rate (GFR) to <60 mL/min per 1.73 m^2^ observed at least twice in not less than 3 months (Azab et al. 2015). Severe loss of renal function and end-stage renal disease (ESRD) are uncommon with lithium treatment, with a prevalence of approximately 1.5%, but 7-folds higher than the general population (Aiff et al. 2015). Risk of renal dysfunction is believed to be associated with longer exposure to lithium as well as with advancing age with or without lithium, and appears to have changed little over the recent decades (Aiff et al. 2015; Jonczyk-Potoczna et al. 2016). Pathological renal changes associated with long exposure to lithium in clinical doses have included the presence of macrocysts, microcysts, glomerulosclerosis, proximal tubular atrophy, and chronic interstitial fibrosis (Albrecht et al. 1980; Oliveira et al. 2010; Alsady et al. 2016; Jonczyk-Potoczna et al. 2016). Molecular mechanisms associated with such dysfunction appear to be multiple and complex. Based mainly on preclinical models, they include alterations in calcium signaling, inositol monophosphate and phosphodiesterase activities, prostaglandins, sodium-solute transport, G-protein-coupled receptors, nitric oxide, vasopressin aquaporin, and inflammation pathways (Rej et al. 2016). However, it is unclear why only some patients develop nephropathy in association with lithium treatment, regardless of age or lithium exposure.

At least 20 findings related to renal dysfunction have emerged from studies of patients treated long term with lithium (Azab et al. 2015). They include (Table 1) (a) significant increase of serum creatinine concentration, not associated with age, in 99 lithium-treated patients followed for up to 10 years (Depaulo et al. 1981); (b) no difference in eGFR among 30 patients aged 55, treated with lithium for 6.2 years and 30 others not exposed to lithium (Hullin et al. 1979); (c) no difference in eGFR among 32 patients aged 49 years, treated with lithium for 5.7 years and 32 matched controls (Bendz 1985); (d) more prevalent low eGFR in 13 patients (mean age, 59 years) treated with lithium for 18 years (5/13) than that in 13 matched controls never exposed to lithium (0/13; *χ* ^2^ = 6.19, *p* = 0.01) (Bendz et al. 1996); (e) no difference in eGFR in 107 patients aged 39 treated with lithium for 4.5 years (eGFR = 86.5 [CI 82.4–90.6]) compared with 29 matched controls (83.9 [76.1–91.7] units) (Coşkunol et al. 1997); (f) lower eGFR among 10 patients/group of average age 35, exposed to lithium for 6.7 years (72.8 [50.7–94.9]) compared to those exposed for 1.3 years (150 [129–172]) or no exposure (125 [112–138] units) (Turan et al. 2002); (g) a risk of ESRD of 0.53% among 3369 subjects of average age 65 exposed to lithium for 23 years, compared to 0.082% of the general Swedish population—a 6.5-fold difference (*χ* ^2^ = 82.5, *p* < 0.0001) (Bendz et al. 2010); (h) eGFR < 60 units in 23.0% of 80 patients treated with lithium up to 38 (mean, 17) years, and more often among men (38%) than women (16%; *p* = 0.04) (Rybakowski et al. 2012); (i) eGFR values were lower in 27.3% of 139 lithium-treated patients of mean age 54, exposed to lithium for ≥1 year, compared to 5.71% among 70 psychiatric controls—a difference of 4.8-fold (*χ* ^2^ = 9.66, *p* < 0.002), and were more likely among older patients and men (Bocchetta et al. 2013); (j) mean GFR 8.0% lower among 330 general practice patients taking lithium compared with 659 matched controls, with similar prevalence of eGFR values ≤60 units in both groups (17.0 vs. 13.1%; *χ* ^2^ = 2.75, *p* = 0.10) (Minay et al. 2013); (k) the rate of dialysis treatment or renal transplantation in the Swedish general population was 0.019%, compared to a 7.8-fold higher rate of 1.5% among 1995 lithium-treated patients of age 66 years given lithium for 27 years (*χ* ^2^ = 176, *p* < 0.0001) (Aiff et al. 2014); (l) no difference in renal function in a 2-year randomized control trial (RCT) for patients given lithium >4 years (Aprahamian et al. 2014); (m) eGFR < 60 units in 12.3% of 2496 general practice patients given lithium for undefined times, compared to a 3.25-fold lower risk of 3.78% in 3864 bipolar disorder patients not given lithium, all of average age 49 (*χ* ^2^ = 165, *p* < 0.0001) (Close et al. 2014); (n) values of eGFR < 60 units were encountered in 32% of 630 subjects aged 66 years treated with lithium ≥10 years, and 4.5% developed ESRD (stage 4 or 5; eGFR < 30 units), with little sex-difference in either outcome (Aiff et al. 2015), (o) eGFR < 60 units was found in 12% of 953 patients given lithium for 10 years and in 50% by 25 years (Bocchetta et al. 2015); (p) no significant difference in the annual decline of eGFR in a case–control study: 305 patients aged 43 given lithium for an average of 4.6 years and 815 controls given other treatments (1.3 vs. 0.9 units) after adjustment for age, baseline eGFR, comorbidities, exposure to nephrotoxic drugs, and episodes of acute lithium toxicity (Clos et al. 2015); (q) eGFR < 60 units was 1.21-times more prevalent among 4678 lithium-treated subjects than among 689,228 controls of mean age 52 treated for up to 28 years, after adjustment for age, sex, and diabetes (estimates: 62.3 vs. 51.4%; *χ* ^2^ = 118, *p* < 0.0001) (Shine et al. 2015); (r) risk for eGFR < 60 units among 3850 patients, aged 54 years, treated with lithium for an average of 1.4 years was 25.7%, and was higher with multiple daily doses, higher serum concentrations, and co-treatment with first-generation neuroleptics (Castro et al. 2016); [s] eGFR < 60 units was about twofold more prevalent among 2148 lithium-treated patients than among those treated with valproate (*n* = 1670), olanzapine (*n* = 1477), or quetiapine (*n* = 1376) (Hayes et al. 2016); (t) in a nationwide population study, clinically diagnosed CKD was increased by up to 3.6-fold with longer exposure to lithium, and associated with use of anticonvulsants (with risk of confounding by selective avoidance of lithium with renal failure), but not antidepressants or antipsychotics (Kessing et al. 2015). To summarize, all these studies point out that lower eGFR is associated with older age and longer exposure to lithium (Table 1).

**Table 1:** Reports on renal effects of lithium treatment

Report	Subjects (n)		Age (years)	Lithium exposure (years)	Main findings
	Li	No Li			
Hullin et al. (1979)	30	30	55	6.2	No difference in eGFR
Depaulo et al. (1981)	99	0	41	2.8	Creatinine increased with Li
Bendz (1985)	32	32	49	5.7	No difference in eGFR
Bendz et al. (1996)	13	13	59	18.0	eGFR fell with Li
Coşkunol et al. (1997)	107	29	39	4.5	No difference in eGFR
Turan et al. (2002)	10	10	35	1.3 and 6.7	eGFR fell with long-term Li
Bendz et al. (2010)	3369	Genl. pop.	65	23.0	ESRD 6.5-fold more often with Li
Rybakowski et al. (2012)	80	0	60	16.0	eGFR < 60: 22.5%; 2.4-times more in men
Bocchetta et al. (2013)	139	70	54	>1.0	eGFR < 60: 4.8-fold more often with Li
Minay et al. (2013)	330	659	48	–	eGFR < 60: similar with/without Li
Aiff et al. (2014)	1995	0	66	27.0	ESRD 7.8-fold more often with Li
Aprahamian et al. (2014)	32	27	74	4.0	No difference in renal function
Close et al. (2014)	2496	3864	49	–	eGFR < 60: 3.25-times less with Li
Aiff et al. (2015)	630	0	66	≥10.0	eGFR < 60: 32%; ESRD: 4.5-fold more with Li
Bocchetta et al. (2015)	1953	0	–	10 and 25	eGFR < 50: 12% in 10, 50% in 25 yrs of Li
Clos et al. (2015)	305	815	43	4.6	No difference in eGFR
Shine et al. (2015)	4678	689,228	52	≤28.0	eGFR < 60: 1.21-fold more often with Li
Castro et al. (2016)	3850	0	54	1.4	eGFR < 60: 25.7% lower with multiple doses/day
Hayes et al. (2016)	2148	4523	46	18	eGFR < 60: ~twofold higher HR with Li
Kessing et al. (2015)	Natl. sample	0	–	–	Clinical CKD 3.6-times more with Li
*N* = 20 studies	>22,296	>699,300	53.1 ± 10.5	10.9 ± 8.9	Function decreased in 15/20 reports (75.0%)

Abnormal renal functioning was associated with longer exposure to lithium in these studies (15.3 ± 9.54 vs. 5.00 ± 0.91 years, respectively [*t* = 2.37, *p* = 0.035])

In addition to changes in renal functioning, the presence of macrocysts or microcysts as possible precursors of loss of kidney function has been reported in several renal-imaging studies following long-term lithium treatment (Tuazon et al. 2008; Slaughter et al. 2010; Farshchian et al. 2013; Karaosmanoglu et al. 2013; Jonczyk-Potoczna et al. 2016). Also, an increase of renal neoplasia during long-term treatment with lithium has been suggested (Zaidan et al. 2014), but not supported by other observations (Baldessarini and Tondo 2014; Licht et al. 2014; Pottegård et al. 2016).

To extend the preceding findings, we evaluated effects of lithium on GFR and other metabolic parameters in a composite sample of 312 bipolar disorder patients followed for 8–48 years in 12 international specialized mood-disorder clinics with extensive experience in the clinical use of lithium.

## Methods

This international collaborative study involved data provided by 12 sites in Argentina, Canada, Germany, Italy, Poland, Spain, and Switzerland (Table 2). Subjects were adults meeting DSM-IV diagnostic criteria for bipolar I or II disorder. Participation was based on meeting local institutional requirements for the ethical conduct of research. Measurements considered included age; sex; years of exposure to lithium treatment; mean daily dose of lithium carbonate and mean daily trough serum concentration of lithium; body-mass index (BMI), white blood cell counts (WBC), and assays of serum concentrations of glucose, blood urea nitrogen (BUN), and creatinine, with estimated GFR (eGFR, in units of mL/min/1.73 m^2^) computed according to the chronic kidney disease (CKD)-epidemiology collaboration (CKD-EPI) formulas for Caucasian (as all study subjects were) women and men (Levey et al. 2009):

**Table 2:** Subject age and lithium exposure across study sites

Study site	Subjects (*n*)	Intake age^a^	Final age^b^	Years on Li^c^
Barcelona: University of Barcelona	26	32.3 ± 8.06	51.8 ± 11.4	19.4 ± 7.41
Berlin: Charité Medical Center	30	39.5 ± 12.4	56.6 ± 14.9	17.2 ± 8.12
Buenos Aires: Palermo University	9	47.2 ± 12.0	62.3 ± 13.4	15.1 ± 5.93
Cagliari: Lucio Bini Mood Disorder Center	50	38.1 ± 12.1	54.6 ± 14.5	17.5 ± 8.61
Cagliari: University of Cagliari	30	37.1 ± 11.2	62.2 ± 13.1	24.1 ± 8.86
Dresden: University of Dresden	22	33.5 ± 12.7	53.8 ± 13.2	20.3 ± 9.49
Halifax: Dalhousie University	27	43.5 ± 13.7	55.4 ± 13.7	11.9 ± 3.97
Lugano: Viarnetto Clinic	21	33.8 ± 11.6	52.0 ± 12.9	18.2 ± 9.45
Pisa: University of Pisa	25	37.6 ± 13.1	49.4 ± 13.8	11.7 ± 6.29
Poznan: University of Poznan	20	43.1 ± 14.2	66.2 ± 10.7	23.1 ± 8.59
Rome: Lucio Bini Mood Disorder Center	46	36.8 ± 15.6	54.6 ± 15.9	18.4 ± 9.47
Würzburg: University of Würzburg	6	45.0 ± 14.9	61.8 ± 9.75	6.80 ± 7.94
Total [95% CI]	312	37.9 [37.5–39.3]	55.8 [54.2–57.4]	17.9 [16.9–18.9]

Total exposure = 6142 person-years

Across the 12 sites, among 312 subjects: ^a^ *t* = 1.55, *p* = 0.007; ^b^ *t* = 1.70, *p* = 0.001; ^c^ *t* = 1.13, *p* = 0.24

$${\text{females}}:{ 141} \times \left( {\left[ {\text{creatinine}} \right]/0. 7} \right)^{ - 0. 3 2 9} \times \left( {\left[ {\text{creatinine}} \right]/0. 7} \right)^{ - 1. 20 9} \times 0. 9 9 3^{\text{age}} \times 1.0 1 8;$$

$${\text{males}}:{ 141} \times \left( {\left[ {\text{creatinine}} \right]/0. 9} \right)^{ - 0. 4 1 1} \times \left( {\left[ {\text{creatinine}} \right]/0. 9} \right)^{ - 1. 20 9} \times 0. 9 9 3^{\text{age}} \times 1.0 1 8.$$

Metabolic measures at baseline were compared over times of exposure to lithium treatment, ranging from 8 to 48 years, using ANOVA methods (*t*-scores). Additional analyses focused on the prevalence of renal dysfunction based on low eGFR (<60 mL/min/1.73 m^2^), and considered standard functional staging, as: *Stage 1* normal functioning (GFR **≥** 90); *Stage 2* mildly decreased functioning (GFR = 60**–**89); *Stage 3* moderate dysfunction (GFR = 30**–**59); *Stage 4* severe dysfunction (GFR = 15**–**29); and *Stage 5* kidney failure (GFR < 15 or needing dialysis) (American National Kidney Foundation, NKF 2002, 2014). Low eGFR included *Stages 3* and *4*.

We addressed the prevalence of low values of eGFR across study sites, and changes with time and in association with selected measures, including ages (at onset, at lithium start and at the last follow up visit), sex, co-occurring medical illnesses, and exposure to lithium (by daily dose, mean serum concentration, and time) as well as to other psychotropic drugs (anticonvulsants, antidepressants, antipsychotics). Associations of potential risk factors were tested by comparing subjects meeting the criterion of at least one low value of eGFR (<60 units) or not, in bivariate comparisons using ANOVA methods (*t*-scores) for continuous measures and contingency tables (*χ* ^2^) for categorical measures, followed by multivariable logistic regression modeling. In order to differentiate effects on eGFR of age and lithium exposure, we also sampled subjects matched for long-term lithium exposure (20–25 years), but starting treatment at ages <40 vs. ≥40 years. Data are shown as mean ± standard deviation (SD) or with 95% confidence interval (CI), unless stated otherwise. Analyses employed commercial software: Statview.5 (SAS Institute, Cary, NC, USA; for spreadsheets), and Stata.12 (StataCorp, College Station, TX, USA).

## Results

### Subject characteristics

The pooled study sample consisted of 312 adult, bipolar disorder subjects, treated with lithium carbonate for 8–48 (mean 17.9 ± 8.62) years (with or without other treatments), representing a total exposure of 6142 person-years. Selected characteristics of subjects from each site (subject count, age at entry to study site, age at last contact, and years treated with lithium) are summarized in Table 2. The proportion of women/men was 57.7/42.3%; age at study site intake averaged 37.9 ± 12.9 (range 11–76) years, and last age averaged 55.8 ± 14.2 (range 20–89) years. Diagnoses were 78.2% bipolar I and 21.8% bipolar II, with an average age at illness-onset of 28.5 ± 11.1 years.

### Metabolic parameters at baseline and during lithium treatment

Summary data for average measures and their status across years of treatment with lithium are based on 2669 assays (Table 3). They include: lithium carbonate dose (833 ± 311 mg/day), mean daily trough serum concentrations of lithium (0.656 ± 0.184 mEq/L), body-mass index (BMI, 27.0 ± 4.86 kg/m^2^), serum glucose concentration (97.3 ± 28.4 mg/dL), blood urea nitrogen (BUN, 26.1 ± 12.9 mg/dL), serum creatinine concentration (0.92 ± 0.24 mg/dL), and estimated glomerular filtration rate (eGFR; 83.3 ± 21.8 mL/min/1.73 m^2^). In addition, white blood cell count ([WBC] not shown) was initially 7.29 and finally 7.59 × 10^−3^/µL, without appreciable change over years of lithium treatment.

**Table 3:** Metabolic parameters in lithium-treated bipolar disorder patients

Exposure (years)	*N*	Lithium dose (mg/day)	Serum [Li^+^] (mEq/L)	BMI (kg/m^2^)	Glucose (mg/dL)	BUN (mg/dL)	Creatinine (mg/dL)	eGFR (mL/min/1.73 m^2^)
Baseline	312	797 ± 283	0.594 ± 0.201	25.9 ± 4.81	88.1 ± 14.3	23.7 ± 11.4	0.87 ± 0.19	94.2 ± 23.3
1	312	859 ± 282	0.638 ± 0.171	27.9 ± 5.87^a^	90.5 ± 16.3	22.1 ± 10.4	0.86 ± 0.17	94.8 ± 22.2
2–5	1561	877 ± 296^a^	0.658 ± 0.185	27.3 ± 5.10^a^	90.9 ± 17.9	23.8 ± 10.8	0.87 ± 0.17	91.2 ± 21.1
6–10	1490	875 ± 310^a^	0.656 ± 0.177	27.5 ± 5.15^a^	97.4 ± 28.6^a^	24.5 ± 11.2	0.90 ± 0.19	86.1 ± 20.1^a^
11–15	1080	858 ± 320	0.672 ± 0.188	27.0 ± 4.57	95.3 ± 24.0^a^	25.4 ± 11.7	0.91 ± 0.21	83.9 ± 20.2^a^
16–20	739	815 ± 305	0.671 ± 0.191	27.6 ± 4.71^a^	98.6 ± 28.4^a^	26.9 ± 14.1	0.93 ± 0.24^a^	78.8 ± 19.3^a^
21–30	760	716 ± 289^a^	0.651 ± 0.172	27.6 ± 4.30^a^	103.8 ± 33.5^a^	31.2 ± 15.7^a^	0.98 ± 0.29^a^	72.7 ± 20.1^a^
≥31	201	647 ± 334^a^	0.607 ± 0.168	26.6 ± 5.03	121.9 ± 54.9^a^	33.1 ± 15.8^a^	1.17 ± 0.48^a^	62.2 ± 22.9^a^
Means [95% CI]	312 subjects	833 ± 311 [824–842]	0.656 ± 0.184 [0.650–0.662]	27.0 ± 4.86 [26.5–27.5]	97.3 ± 28.4 [94.1–100.5]	26.1 ± 12.9 [24.7–27.5]	0.92 ± 0.24 [0.89–0.95]	83.3 ± 21.8 [80.9–85.7]
Change (%/year)	2669 assays	–0.777 [–0.654 to –0.901]	–0.005 [–0.118 to –0.118]	+0.162 [0.011–0.311]	+0.787 [0.619–0.954]	+1.41 [1.10–1.72]	+0.724 [0.609–0.839]	–0.915 [–0.822 to –1.01]
*p* value [*t*-score]	2669 assays	<0.0001 [12.4]	0.94 [0.077]	0.04 [2.11]	<0.0001 [9.21]	<0.0001 [8.91]	<0.0001 [12.4]	<0.0001 [19.1]

Data are based on means of *N* measurements (of a total of 2669) for 312 subjects over stated exposures to lithium treatment

Not shown are data for white blood cell counts (WBC), which did not change appreciably (initital: 7.29, final: 7.59 × 10^–3^ per µL)

*BMI* (body-mass index [kg/m^2^]), *BUN* (blood urea nitrogen [mg/dL]), *creatinine* [mg/dL], *eGFR* (estimated glomerular filtration rate for creatinine [mL/min/1.73 m^2^]), *glucose* (not necessarily fasting [mg/dL]), *WBC* (white blood cell count [thousands/µL])

^a^Values differ significantly from baseline measure, based on Tukey–Kramer post hoc tests comparing each exposure-interval to baseline values

The dose of lithium carbonate declined significantly over the years (0.78%/year) whereas serum lithium concentrations remained stable over time, reflecting dosing adjustments as lithium clearance decreased with advancing age (Table 3). BMI increased slightly, from 25.9 initially to a final mean value of 26.6 kg/m^2^, at a rate of 0.16%/year, and did not differ significantly between those given psychotropic drugs other than lithium or not (27.0 ± 5.36 vs. 26.3 ± 3.72 kg/m^2^; *t* = 1.36, *p* = 0.18). Serum glucose concentration also rose significantly over years of treatment and with advancing age, at 0.79%/year. BUN increased appreciably (from 23.7 to 33.1 mg/dL), at a rate of 1.4%/year, and creatinine rose at about half the rate of BUN, from 0.87 to 1.17 mg/dL, at 0.72%/year.

The metabolic measure of particular interest, eGFR, declined from a mean at intake of 94.2, to a final average of 62.2 mL/min/1.73 m^2^, at an average rate of decline of 0.915%/year (Table 3). Times to first-observed significant increases in various measures vs. baseline included: *BMI*, just after the first year; *glucose*, from years 6–10; *creatinine*, years 16–20; and *BUN*, years 21–30. Decline of eGFR was noted starting from years 6–10, with a projected decline to the lower limit of normal (60 units) after 30 or more years of exposure to lithium (Table 3).

### Staging of renal deficiency

**Fig. 1:**
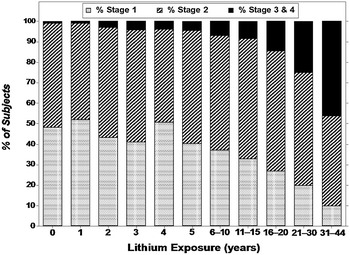
Proportion (%) of subjects with *Stages 1, 2*, or *3* and *4* of renal function vs. years of lithium exposure. By linear regression, the prevalence of *Stage 1* renal function (normal) declined highly significantly (slope [*β*]: −1.12 [CI −1.36 to −0.88]; *t* = 10.6, *p* < 0.0001); *Stage 2* (mild dysfunction) remained stable (*β*: –0.035 [CI −0.373 to 0.303], *t* = 0.23, *p* = 0.82); and *Stages 3* + *4* increased highly significantly (*β*: +1.15 [CI 0.91–1.40], *t* = 10.7, *p* < 0.0001)

We considered changes in the prevalence of stages of renal function based on standard values (American National Kidney Foundation, NKF 2002, 2014) of eGFR, vs. years of lithium treatment (Fig. 1). The prevalence of normal or *Stage I* eGFR (≥90 mL/min/1.73 m^2^) declined over years of lithium exposure (and advancing age), from 48.3% at intake to 9.80% after 31–48 years of exposure, whereas *Stage 2* (60–89 units) declined slightly (from 50.8 to 44.1% of subjects), and the prevalence of abnormal eGFR *Stages 3 and 4* eGFR (15–59 units) increased from 0.85 to 46.1%. No subject reached end-stage renal failure (*Stage 5*). The relative risk of each stage by sex (women/men) was: *Stage 1*, 0.72 (more in men); *Stage 2*, 1.11; *Stage 3*, 1.68; and *Stage 4*, 9.29 (all three more in women); these sex-differences were highly significant (overall *χ* ^2^ [d*f* = 3] = 51.3, *p* < 0.0001).

### Comparison of subjects with low vs. normal eGFR

A total of 92 (29.5%) of the 312 subjects had at least one estimate of eGFR below the lower limit of normal (<60 mL/min/1.73 m^2^). Based on the widely accepted criterion of ≥2 low values (Azab et al. 2015), the risk of eGFR < 60 units was 18.1% [CI 13.9–22.7]. Characteristics of *subjects* with vs. without low eGFR values were compared in initial bivariate comparisons (Tables 4, 5). Subjects with significantly lower eGFR were (a) more often women than men; (b) older at illness-onset, at starting lithium, and at final observation; (c) significantly less likely to be given co-treatments with anticonvulsants or antipsychotics; and (d) having significantly lower initial values of eGFR (Table 4). In addition, measures associated with low eGFR based on all *assays*, included (e) older at the time of assays; (f) more likely to have medical comorbidities (mainly cardiovascular diseases, diabetes, hypercholesterolemia, hypertension, hypertriglyceridemia, hypothyroidism, or respiratory diseases); (g) longer exposure to lithium; (h) *lower* average doses of lithium carbonate; (i) without differences in mean serum lithium concentrations; (j) higher mean BUN; (k) higher serum glucose concentration; and (l) higher BMI (Table 5). Factors *not* associated with low eGFR included (a) diagnosis; (b) educational level; (c) metabolic syndrome; (d) abuse of alcohol or drugs; (e) cigarette smoking; (f) lifetime suicidal behavior, and (g) serum TSH levels.

**Table 4:** Factors associated with vs. without low eGFR among 312 bipolar disorder subjects treated long-term with lithium

Factor	Low eGFR^a^	Normal eGFR	*p*-value [*χ* ^2^ or *t*-score]
Subjects	92	220	–
Sex (%)			0.003 [8.98]
Women	36.1	63.9	
Men	20.5	79.5	
Ages (years)			
Illness onset	31.8 [29.4–34.2]	27.1 [25.7–28.5]	0.0007 [3.41]
Started lithium	42.5 [39.8–45.2]	35.9 [34.2–37.6]	<0.0001 [4.19]
Final lithium	65.0 [62.4–67.6]	52.0 [50.3–53.7]	<0.0001 [8.09]
Co-treatments (%)			
Anticonvulsants	23.7	45.5	0.001 [10.4]
Antipsychotics	47.4	64.6	0.01 [6.40]
Antidepressants	25.0	36.1	0.07 [3.25]
Initial eGFR	77.2 ± 16.1	94.6 ± 21.9	<0.0001 [6.74]

Means are with 95% CI. Serum lithium concentration is in mEq/L; dose is of lithium carbonate is total mg/day. Additional factors *not* associated with low eGFR: (1) diagnosis (bipolar I vs. bipolar II), (2) education, (3) metabolic syndrome (overall risk = 30.4%), (4) any substance abuse, (5) alcohol abuse, (6) smoking, (7) any suicidal act, (8) serum TSH. Medical illnesses include cardiovascular and metabolic syndromes

^a^Low eGFR: subjects with at least one value <60 mL/min/1.73 m^2^; the observed rate of such subjects was 92/312 (29.5%), but 312/2669 assays (11.3%)

**Table 5:** Measures associated with vs. without low eGFR among 2669 assays in 312 bipolar disorder subjects treated long-term with lithium

Measure	Low eGFR^a^	Normal eGFR	*p*-value [*χ* ^2^ or *t*-score]
Age at assay	62.7 [61.4–64.0]	48.0 [47.5–48.5]	<0.0001 [18.3]
Medical comorbidity (%)^b^	83.5	59.5	<0.0001 [41.3]
Lithium exposure			
Years treated	19.6 [18.5–20.7]	11.2 [10.9–11.5]	<0.0001 [16.3]
Mean dose	588 [554–622]	884 [871–896]	<0.0001 [15.5]
Mean serum [Li+]	0.65 [0.63–0.68]	0.66 [0.65–0.67]	0.32 [0.99]
Physiological measures			
BUN	36.7 [34.3–39.1]	24.6 [24.0–25.2]	<0.0001 [12.6]
[Glucose]	108 [103–112]	95.8 [94.4–97.2]	<0.0001 [5.88]
BMI	28.5 [26.2–30.8]	26.4 [25.9–26.9]	0.03 [2.22]

Means are with 95% CI. Serum lithium concentration is in mEq/L; dose is of lithium carbonate is total mg/day. Additional factors *not* associated with low eGFR: (1) diagnosis (bipolar I vs. bipolar II), (2) education, (3) metabolic syndrome (overall risk = 30.4%), (4) any substance abuse, (5) alcohol abuse, (6) smoking, (7) any suicidal act, (8) serum TSH. Medical illnesses include cardiovascular and metabolic syndromes

^a^Low eGFR: subjects with at least one value <60 mL/min/1.73 m^2^; the observed rate of such subjects was 92/312 (29.5%), but 312/2669 assays (11.3%)

^b^Most frequent medical comorbidities are: cardiovascular diseases, diabetes, hypercholesterolemia, hypertension, hypertriglyceridemia, hypothyroidism, and respiratory diseases

Of note, antipsychotic drugs (59.2% of all subjects) were given with lithium more than either anticonvulsants (38.6%) or antidepressants (33.1%). Based on multiple variable logistic regression modeling, adjusted for age and sex (not shown), mood-altering anticonvulsants were associated with shorter exposure to lithium and lower serum concentrations. Use of antipsychotics was significantly greater among subjects diagnosed with bipolar I than II disorder, as well as shorter exposure to lithium but at higher doses and serum concentrations. Antidepressants were given more often to bipolar II than bipolar I disorder subjects.

### Declining eGFR with age and exposure to lithium

**Fig. 2:**
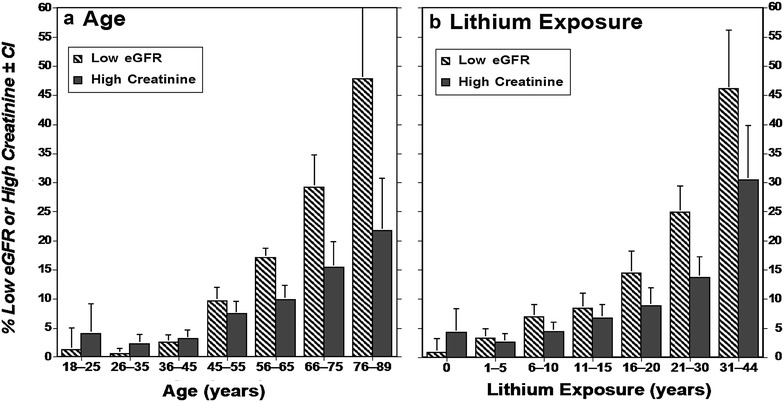
Values of eGFR (mL/min) [with 95% CI] among men and women: **a** vs. age; **b** vs. years of lithium treatment, pooled from 12 international sites, involving 2669 measurements among 312 bipolar disorder subjects treated with lithium for 8–44 years

As expected, advancing age and years of lithium treatment were associated with low values for eGFR (<60 mL/min/1.73 m^2^), with corresponding increases in rates of elevated serum creatinine concentration (defined as >1.2 mg/dL, based on the lower standard value for women rather than that of 1.5 mg/dL for men) (Fig. 2; Table 3). The rate of decline (slope function as %/year) averaged 0.710%/year of age, with a nonsignificantly steeper decline among women than men (0.756 vs. 0.631%/year), and 0.915%/year of lithium exposure, with a significantly greater (non-overlapping CIs) rate of decline among women than men (0.934 vs. 0.785%/year of lithium; Table 6). The overall observed rate of decline of eGFR was 28.9% greater for years of lithium treatment than for years of age (0.915 vs. 0.710%/year; Table 6).

**Table 6:** Rate of decrease of eGFR with age and lithium exposure (%/year)

Group	eGFR decrease, %/year [95% CI] (*n*)		
	Age: healthy adults	Age: lithium-treated	Years of lithium
All subjects	0.637 [0.497–0.777] (365)	0.710 [0.653–0.767] (312)	0.915 [0.822–1.08] (312)
Men	0.488 [0.361–0.616] (160)	0.631 [0.545–0.717] (132)	0.785 [0.546–0.717] (132)
Women	0.754 [0.580–0.928] (205)	0.756 [0.682–0.829] (180)	0.934 [0.815–1.05] (180)

Lithium-treated subjects are from the present study. Data for healthy adults are adapted from Rule et al. (2004) for clearance of iothalamate. Rates of GFR decrease as %/year are computed as [initial eGFR − observed eGFR]/[initial eGFR] × 100 with 95% confidence intervals and number (*n*) of subjects (eGFR is in units of mL/min/1.73 m^2^)

For comparison with subjects not exposed to lithium, we obtained data from a study by Rule et al. (2004) who measured the effect of age in 365 healthy subjects on eGFR estimated as in the present study. These data indicate a nonsignificantly lower rate of decline with age without than with lithium treatment (0.637 vs. 0.710%/year), as well as a slightly larger decreases among women than men (Table 6). In addition, the rate of decline of eGFR was significantly greater with years of exposure to lithium (0.915%/year) than with age in subjects not exposed to lithium (0.637%/year; Table 6).

### Renal effects of age at starting lithium treatment

Given the uncertain relative contributions of aging and exposure to lithium on declining eGFR, we considered a restricted sample of 610 assays, matched for long-term treatment with lithium for 20–25 years (mean for both = 22 years), but starting treatment at ages <40 vs. ≥40 years (Table 7). Mean eGFR was highly significantly lower among participants starting lithium at age ≥ 40 years. Moreover, risk of low values of eGFR was nearly twice as high (1.94-times) among the older subjects, despite similar exposure to lithium.

**Table 7:** Patients started on lithium treatment below or above age 40

Measure	Age < 40	Age ≥ 40	*p*-value (*t* or *χ* ^2^)
Subjects (*n*)	426	184	–
Years of lithium	22.3 ± 1.69	22.1 ± 1.63	0.123 (0.35)
Mean eGFR	77.6 ± 19.9	68.3 ± 17.9	<0.0001 (4.02)
eGFR < 60 units (%)	37.1	72.3	<0.0001 (63.8)

### Multivariable regression modeling

Finally, we carried out multivariable logistic regression modeling of factors associated with low eGFR (<60 mL/min/1.73 m^2^). Factors remaining independently and significantly associated ranked: (a) longer treatment with lithium, (b) lower mean daily dose of lithium carbonate, (c) higher mean serum lithium concentration, (d) older age at the time of assays, (e) co-occurring medical illness (Table 8). Not significantly associated with low eGFR in such modeling were sex, BMI, and co-treatment with an anticonvulsant, antipsychotic, or antidepressant drugs. Of note, serum concentration was associated with low eGFR only when adjusted for age, dose, and duration of exposure (Table 8), but not without such adjustment (Tables 4, 5).

**Table 8:** Multivariate logistic regression model for factors associated with low eGFR (<60 mL/min/1.73 m^2^)

Factor	Odds ratio [95% CI]	*z*-score	*p*-value
Longer lithium treatment (years)	1.07 [1.04–1.09]	5.92	<0.0001
*Lower* mean lithium dose (mg/day)	1.003 [1.002–1.004]	5.42	<0.0001
Higher mean serum [Li^+^]	4.47 [4.36–43.5]	4.47	<0.0001
Older age at assay	1.04 [1.02–1.06]	4.32	<0.0001
Co-occurring medical illness	2.11 [1.28–3.49]	2.91	0.004

## Discussion

Among 312 adult bipolar disorder patients, treated for 8–48 years with lithium carbonate (6142 person-years of exposure), from 12 international collaborating centers we found an incidence of low eGFR (<60 units) of 18.1% of subjects for ≥2 low values. The risk was 29.5% using a broad criterion of one low value, for which the overall female/male risk ratio was 1.76. *Stage 1* eGFR (values of ≥90 units) was 39% more prevalent among men than women, whereas *Stages 2* (60–89; by 11%), *3* (30–59; 68%), and *4* (15–29 units, by 9.3-fold) were more frequent among women. No subject reached end-stage renal dysfunction (ESRD), perhaps reflecting the source of study data from specialized mood-disorder clinics where close clinical follow-up would lead to suspension of treatment before reaching ESRD. Close clinical monitoring probably is also reflected in the lack of decline in average serum concentrations of lithium over years of treatment, despite a significant decline in total daily dose, presumably adjusted to maintain stable blood levels.

A particularly important finding is that eGFR declined with longer exposure to lithium treatment, but also with corresponding increases in age (Fig. 2; Table 6). Both factors were sustained as significant and independent in multivariable modeling (Table 8). Effects of aging on renal function are well established even among human subjects without known disease or toxic factors (Rule et al. 2004; Weinstein and Anderson 2010). Although we did not have a comparison sample of patients followed over time without lithium treatment, we could compare rates of decline of eGFR as a function of age and of time of exposure to lithium, and with reported rates of decline in healthy subjects (Rule et al. 2004). Without lithium treatment, the rate of decline in eGFR (%/year) vs. age in normal subjects averaged 0.637 [CI 0.497–0.777] (Rule et al. 2004), compared to 0.710 [0.653–0.767] for age in lithium-treated subjects, and to 0.915 [0.822–1.08] for years of lithium treatment (Table 6). These estimates are similar, with overlapping confidence intervals for the effect of aging, but a higher rate with lithium exposure. Additional reported data indicate a rate of decline of eGFR in healthy subjects of 0.708 [0.644–0.772]  %/year (American National Kidney Foundation, NKF 2014)—a value even closer to that found in our study for age among lithium-treated subjects. We also addressed the relative contributions to declining eGFR by considering a sample of subjects matched for long-term exposure to lithium (22 years), but starting the treatment at ages <40 vs. ≥40 years (Table 7). Mean eGFR was significantly higher among participants who started lithium at older ages, and the risk of low values of eGFR was nearly twice greater among the older subjects, despite similar exposure to lithium. These findings indicate that effects of aging were greater than the exposure to lithium. Moreover, adverse effects of lithium on renal function may be greater at older ages.

The rate of decline of eGFR averaged 0.92% per year of lithium treatment, and was 19% higher among women than men. The observed overall rate of decline is consistent with most (Bendz et al. 1996, 2010; Bocchetta et al. 2013, 2015; Close et al. 2014; Aiff et al. 2015; Shine et al. 2015; Kessing et al. 2015; Hayes et al. 2016), but not all (Clos et al. 2015) retrospective reports on the effects of lithium on kidney function. In the study by Clos et al. (2015), however, patients were exposed to lithium for an average of 55 months, possibly too brief to support detection of effects on kidney function (Davis et al. 2015; Bocchetta et al. 2016). Interestingly, however, a similar lack of effect of lithium on renal function was found in a 4-years prospective study in elderly patients with mild cognitive impairment (Aprahamian et al. 2014). In the present findings, average values of eGFR became significantly lower than baseline levels by 6–10 years of treatment, and a mean decline to the lower limit of normal (60 units) required ≥30 years of exposure to lithium (Table 3). Other reports of long-term lithium treatment effects on renal function are consistent with this observation (Bendz et al. 2010; Bocchetta et al. 2015; Shine et al. 2015). We also found that risk of later low values of eGFR were strongly predicted by lower initial values (Table 4). Exposure to lithium treatment needed to be at least 6–10 years to be associated with significant decreases of eGFR (Table 3).

Our finding of greater risk of a decline in eGFR among women is also consistent with some recent reports (Bocchetta et al. 2015; Shine et al. 2015) suggesting a higher vulnerability of women to developing lithium-related effects on kidney. Of note, however, other studies have found greater risk of declining eGFR among men (Rybakowski et al. 2012; Bocchetta et al. 2013) or no sex difference (Aiff et al. 2015).

Serum concentrations of urea (BUN) increased by 1.41%/year, glucose by 0.787%/year, and creatinine by 0.724%/year—all rising with longer exposure to lithium and correspondingly advancing age. The observed increase in serum glucose levels contrasts with a study reporting a nonsignificant increase in glucose levels after four years of treatment with lithium in elderly patients (Aprahamian et al. 2014). Interestingly, studies in animals evaluating the effects of lithium on glucose metabolism also may be discordant with our findings (Shah and Pishdad 1980; Tabata et al. 1994). Indeed, whereas Shah and Pishdad (1980) found that lithium induced the hyperglycemia in rats, Tabata et al. (1994) found a markedly increased sensitivity of glucose transport to insulin after lithium treatment.

We also found that average BMI increased by 0.162%/year of treatment with lithium, with a significant increase over baseline values by the end of the first year of exposure, but little more thereafter (Table 3). This finding confirms that lithium may contribute to weight gain (Mathew et al. 1989; Atmaca et al. 2002), although the effect might reflect exposure to other weight-increasing agents including antipsychotic drugs (Calkin et al. 2009). However, the potential adverse risks associated with long-term treatment with lithium need to be balanced against major clinical benefits of treatment with lithium (McKnight et al. 2012; Severus and Bauer 2013; Kessing et al. 2015).

Several factors were associated with loss of eGFR during long-term treatment with lithium (Tables 4, 5, 6, 7 and 8). Notably, declining eGFR was associated with serum lithium concentrations only when adjusted for age, dose of lithium, and duration of lithium exposure, whereas total daily doses of lithium carbonate were actually *lower* with low eGFR, in association with older age (Tables 4, 5, and 8). These findings suggest that dose was adjusted to maintain therapeutic serum levels in the face of declining renal clearance of lithium and with age. We also found that medical comorbidities (especially diabetes and hypertension) were associated with declining eGFR. In contrast, use of adjunctive treatments, especially modern antipsychotic drugs and mood-altering anticonvulsants, were associated with *less* risk of low eGFR values (Tables 4, 5). These associations are not readily explained. Patients with low eGFR were older, had more general medical comorbidity, and were given fewer psychotropic drugs of all kinds as well as lower doses of lithium. Of note, there is suggestive evidence that anticonvulsants and antipsychotics may themselves contribute to risk of renal damage (Hwang et al. 2014; Kessing et al. 2015). Other findings implicate episodes of acute lithium intoxication (possibly an indication of more aggressive treatment) with declining renal function (Rej et al. 2012).

There may be effects of once-daily vs. multiple daily dosing with lithium on renal function (Carter et al. 2013; Castro et al. 2016). Some evidence suggests less renal toxicity with once-daily dosing, but the findings are inconsistent, and may be confounded by likely use of lower doses with once-daily regimens (Schou et al. 1982; Carter et al. 2013). Moreover, the observed effects pertain mainly to small reductions in 24-h urine volume (Kusalic and Engelsmann 1996). Once-daily dosing is perhaps best reserved for young, vigorous patients given moderate doses of lithium to limit the potentially toxic impact of high, daily peak serum concentrations. Reducing lithium dose might be expected to limit toxic effects as was supported by present findings (Tables 4, 5, assuming that dose was not lowered because of declining renal function). Lithium dose can be reduced by use of combinations with other agents with mood-stabilizing effects, including some anticonvulsants or antipsychotics.

Consideration of these factors during appropriately close, long-term clinical monitoring should help to limit risks of renal impairment with long-term lithium treatment (Paul et al. 2010). In addition, there may be benefits in monitoring serum concentrations of lithium levels relatively frequently, especially in elderly patients. It has been suggested that lithium levels should be monitored every 3 months since even a single occurrence of a level higher than 1.0 mEq/L may result in a modest but significant decrease of the GFR lasting for at least 3 months (Bauer et al. 2006; Van Beneden et al. 2011; Kirkham et al. 2014; Davis et al. 2015; Shine et al. 2015). In general, we would emphasize the importance of appropriate selection of patients for long-term lithium treatment, maintaining them on minimum effective doses and daily trough serum concentrations especially for older populations, and regular monitoring to assess adherence to prescribed treatment. These principles of safe practice are important to emphasize, especially as many mood-disorder patients are followed by primary-care clinicians and are not followed in specialized programs directed by experts (Müller-Oerlinghausen et al. 2012).

### Limitations

The present findings should be interpreted in the context of some limitations. First, the study is retrospective in nature. However, clinical data were collected longitudinally in specialized mood-disorder clinics where patients are followed up systematically and at regular intervals, increasing the statistical accuracy of gathered information. Second, the main measure of renal function in this study was estimated GFR based on serum concentrations of creatinine, and not on independently verified clearance of an exogenous test molecule. The formulas employed may not adequately reflect the rate of glomerular filtration at very high concentrations of creatinine, such as >1.75 mg/dL (Levey et al. 2009; Stevens 2013). Nevertheless, eGFR is widely employed measure of renal function and is readily obtained for routine clinical use. Finally, we lacked a comparison group without lithium treatment, leaving the important question of effects of aging vs. of lithium on eGFR unresolved.

## Conclusions

This multisite, international, long-term study found significant changes in renal function and other metabolic measures in association with very prolonged treatment with lithium carbonate given to prevent recurrences of bipolar disorder. It adds to evidence that long-term lithium treatment is associated with a decline in renal function as expressed by significant decreases of eGFR over time. We found moderate decreases in eGFR after prolonged exposure to lithium (at least 6–10 years) and advancing age, along with increases in serum creatinine and BUN concentrations, and small increases in glucose levels and BMI. No cases of severe or end-stage renal failure were encountered, probably owing to close clinical monitoring and timely interventions in cases with declining renal function. We found greater decreases in eGFR among women than men, and following lower initial values of eGFR, as well as when lithium treatment was started at older ages. The study findings contribute to clarifying relationships between long-term lithium treatment and its metabolic safety profile. Overall, this study and those summarized above (Table 1) indicate, with some notable inconsistencies, that there were major risks of declining eGFR with very prolonged treatment with lithium salts, but that effects of advancing age also are prominent and confound quantification of risk-by-time specific to lithium exposure. Noteworthy risk factors for low eGFR included female sex, higher serum lithium level, longer lithium treatment, lower initial eGFR, and medical comorbidity, as well as older age. A clinically favorable conclusion is that emerging decreases in renal function can be detected readily with regular metabolic monitoring, and probably modified by timely interventions that include an increasing number of apparently effective treatment options to lithium.
